# Potential of Atlantic Codfish (*Gadus morhua*) Skin Collagen for Skincare Biomaterials

**DOI:** 10.3390/molecules28083394

**Published:** 2023-04-12

**Authors:** Cristina V. Rodrigues, Rita O. Sousa, Ana C. Carvalho, Ana L. Alves, Catarina F. Marques, Mariana T. Cerqueira, Rui L. Reis, Tiago H. Silva

**Affiliations:** 1 3B’s Research Group, I3Bs—Research Institute on Biomaterials, Biodegradables and Biomimetics, University of Minho, Headquarters of the European Institute of Excellence on Tissue Engineering and Regenerative Medicine, AvePark—Parque de Ciencia e Tecnologia, Zona Industrial da Gandra, Barco, 4805-017 Guimarães, Portugal; 2 ICVS/3B’s—PT Government Associate Laboratory, 4806-909 Braga/Guimarães, Portugal

**Keywords:** Atlantic codfish, collagen, membranes, skincare, wound healing, marine biomaterials

## Abstract

Collagen is the major structural protein in extracellular matrix present in connective tissues, including skin, being considered a promising material for skin regeneration. Marine organisms have been attracting interest amongst the industry as an alternative collagen source. In the present work, Atlantic codfish skin collagen was analyzed, to evaluate its potential for skincare. The collagen was extracted from two different skin batches (food industry by-product) using acetic acid (ASColl), confirming the method reproducibility since no significant yield differences were observed. The extracts characterization confirmed a profile compatible with type I collagen, without significant differences between batches or with bovine skin collagen (a reference material in biomedicine). Thermal analyses suggested ASColl’s native structure loss at 25 °C, and an inferior thermal stability to bovine skin collagen. No cytotoxicity was found for ASColl up to 10 mg/mL in keratinocytes (HaCaT cells). ASColl was used to develop membranes, which revealed smooth surfaces without significative morphological or biodegradability differences between batches. Their water absorption capacity and water contact angle indicated a hydrophilic feature. The metabolic activity and proliferation of HaCaT were improved by the membranes. Hence, ASColl membranes exhibited attractive characteristics to be applied in the biomedical and cosmeceutical field envisaging skincare.

## 1. Introduction

Collagen is the most abundant protein in the human body, accounting for approximately 30% of the total body protein weight, meaning about one-fourth of the total protein content in most animals [1,2]. This molecule has a complex supramolecular structure, occurring with diverse morphologies in each tissue, lending it to a range of biological functions. Collagen participates in diverse processes in the organism such as helping the formation of the extracellular matrix (ECM), which is accomplished by its deposition, among other components, by fibroblasts. Collagen also maintains specific interactions with different receptors, for instance integrins and some glycoprotein, being part of the signaling pathway for cell adhesion, migration, differentiation, growth and survival, among others [1,2,3,4,5]. To date, 28 types of collagen were identified and characterized, presenting a triple helix structure [6,7]. Although all collagen types share this type of conformation, their structures display some variations, which gives them specific functions in the organism. From all these collagen types, the most abundant in the human organism is the type I, representing more than 90% of the total collagen. Type I collagen is composed by three α chains: two identical α1 chains and an α2, that merge together to create the typical triple-helix conformation [2,3,8,9]. Each α chain is composed by a repeating sequence of three amino acids (Gly-X-Y), in which glycine (Gly) is always present, and usually proline (Pro) and hydroxyproline (OHyp)—with the latter being exclusive of collagens. It is also common in this type, mainly in older animals, that the α chains associate through covalent bonds, forming macromolecules with higher molecular weight, denominated β and ϒ components [2,6,10].

Different strategies have been studied for the production of collagen, mostly based in isolation from animal tissues, but also exploring heterologous expression, with certain attention being given in the latest years to aquatic organisms, explored as a source not only of collagen [11,12], but of many other bioactive compounds. Furthermore, some studies reveled that marine type I collagen can be safer than the mammalian one, since it cannot transmit zoonotic diseases and is highly biodegradable. Besides, it has no religious or cultural limitations and can be easily extracted with high yields and low production costs, depending on the species and methodology used. Since type I collagen can be extracted from diverse fish parts, its industrial application can represent a useful approach to valorize by-products (skin, bones, muscles, scales and swim bladders) resulting from their processing for food [3,4,8,11,13,14,15]. In particular, the Atlantic codfish, *Gadus morhua*, plays a major role in the fisheries and economy all over the world, whereby its capture and production represented 1,303,770 tons in 2017 [2,16,17,18,19]. The codfish skin, specially, is one of the main by-products resulting from the fish shredding or fillets production, and it can be used for the extraction of collagen to produce gelatin or hydrolysates with high yields [20]. Thus, marine collagen, particularly the one from Atlantic codfish skin, seems to be a reliable and safe substitute (generally recognized as safe by the Food and Drug Administration) for the mammalian extracted collagen [21]. Hence, this specific collagen deserves to be further studied and evaluated in a way to determine its characteristics, biological activities and possible applications for the industry.

Once type I collagen is so abundant in the ECM of human skin, maintaining its structure and function, it is commonly used for both biomedical and cosmetic applications [2,22]. Due to the numerous potential applications of collagen, its market has been growing due to the increasing demand for biomaterials with those functions, being estimated to achieve up to USD 6.63 billion by 2025 [4,16,17,23,24,25]. Moreover, the use of marine collagen derivatives on biomaterials has already shown positive results for wound healing, helping the proliferation of skin cells such as keratocytes [22,26]. The type of collagen derivatives can be classified based on their degree of hydrolysis, and are usually divided into undenatured collagen, gelatin and collagen hydrolysates. These derivatives can be used in various ways as scaffolds to promote the simulation of the natural environment of the ECM for tissue engineering and regeneration, as bioactive agents to influence cell behavior (e.g., promoting skin cells proliferation) or even as a drug delivery system [9,13,16,27]. Collagen, being a natural component with a high degree of structural order and stiffness, is considered to be an ideal choice for the development of those scaffolds, for further application in tissue engineering and drug delivery systems. In particular, collagen membranes have been proposed, based in collagen alone or in combination with other components (e.g., hyaluronic acid, chitosan, elastin, alginates). This type of biomaterial can be achieved through different methods such as solvent casting [28], electrospinning [29] and spin-coating [30]. Moreover, they can be formulated with the additional capability to deliver therapeutic compounds such as growth factors or antimicrobials, functioning as a bioactive dressing. They can also help to moisturize the skin surface and absorb the wound exudate excess and degraded components from the damage ECM, as well as growth-inhibiting molecules such as proteases, cytokines, and free radicals, while protecting the wound from trauma and microorganisms, allowing gaseous exchanges [28,31,32,33]. Additionally, because of the gradual removal of excess water from the wound by the biomaterials, the type I collagen fibrils become stiffer, giving more resistance to the newly formed ECM [34]. In this way, these collagen biomaterials promise to help maintain the physiological morphology of the cells, support the growth of epidermal cells, prevent collagen degradation by collagenase activity and reduce reactive oxygen species (ROS), providing structural integrity to the damaged tissue [33,34,35,36,37]. Hence, scaffolds such as collagenous membranes are ideal to be applied in skincare [28,29].

The main objective of this work was to valorize the Atlantic codfish by-products, namely skin, through the evaluation of its type I collagen for skincare applications. The isolation of collagen from *Gadus morhua* skin, obtained from fish processing industries, as well as its physicochemical characterization were made taking the homologous protein from bovine as reference, to assess biochemical differences. In a biomedical and cosmeceutical perspective, it was meant for the development of a codfish collagen-based membrane for wound dressings, with in vitro assessment using keratinocyte cell line.

## 2. Results and Discussion

### 2.1. Collagen Extraction and Characterization

#### 2.1.1. Yield of Extraction

A total of six extractions of collagen from two batches of Atlantic codfish skins (collected in 2018 and 2019) were completed successfully by acid extraction. Minor differences were observed between the extraction yields, as shown in Table 1, with an obtained yield of 3.57% ± 0.70% for the 2018 batch and of 4.43% ± 0.32% for the 2019 batch. The variations in yields of different batches are statistically non-significant (*p* ≥ 0.05), confirming the method’s reproducibility for the extraction of collagen from codfish skins, based on the acetic acid extraction method. The reported yields of collagen extraction from other by-products and species, such as codfish swim bladders and seaweed pipefish skin, using the same method of extraction, were 5.72% [38] and 5.5% [12], respectively. These small variations might be due to the different collagen structures between by-products and species, for instance, within the crosslinking of the collagen fibrils [39]. Moreover, the treatment of codfish with salt brine can also influence the properties of the resulting skins, namely the moisture contents, thus affecting the accuracy on the determination of the extraction yield. For instance, Bisht et al. [40] used deep eutectic solvents for the extraction of type I collagen from the skin of *Gadus morhua,* obtaining a yield of 6%, when calculated on a dry weight basis. Moreover, Skierka et al. [41] obtained distinct yields, calculated based on hydroxyproline quantification, when extracting collagen from the skin of the same species by acid or enzymatic extraction (yield variations between 18–90%, but in respect to the initial collagen contents in fish skin). Nevertheless, values of collagen mass of up to 10% of skins weight are according to the ones found in the literature for several fish species and characterize an efficient extraction method [42].

#### 2.1.2. Molecular Profile

**Figure 1:**
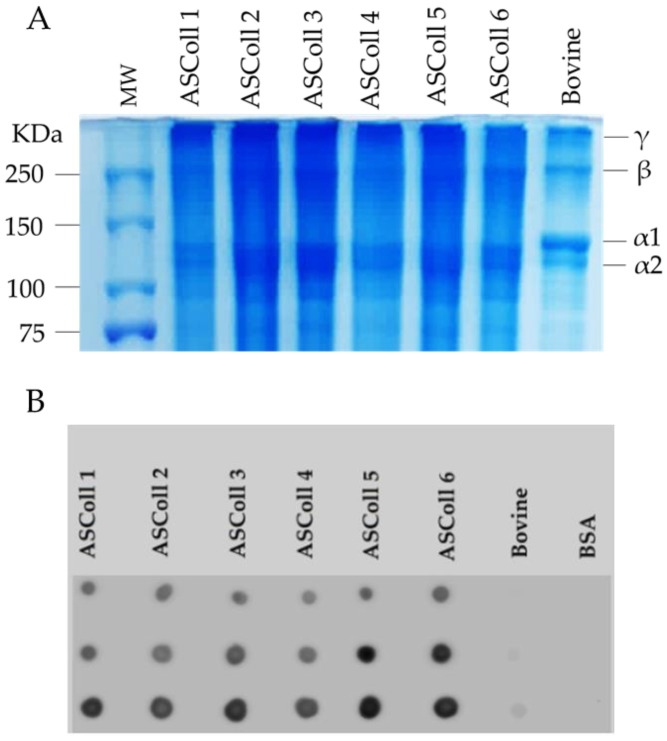
Molecular profile of ASColl. (**A**)—Electrophoresis profile from the ASColl samples and type I collagen from bovine skin. MW—molecular weight standard protein marker. (**B**)—Dot blot analysis of the ASColl samples and type I collagen from bovine skin. BSA—Bovine serum albumin; bovine—type I collagen from bovine skin.

In order to analyze the collagen extracted from Atlantic codfish skin and to assess its respective molecular weight, the ASColl samples were separated by SDS-PAGE, and compared with the pattern from a commercial bovine skin type I collagen sample. As shown in Figure 1A, the characteristic type I collagen bands can be identified in all samples. The α1 and α2 chains are found with molecular weights between 100 kDa and 150 kDa, while the β and ϒ chains are found above 250 kDa, which corresponds to dimers and trimers of the α chains, respectively, being in agreement with previous studies in other species [39,43]. Moreover, the SDS-PAGE profile is identical between every ASColl sample, supporting the hypothesis of identical structures between ASColl samples from different extractions, indicating a reproducible extraction method and equivalent codfish skin batches. Moreover, the electrophoretic profiles of the ASColl samples are similar to the one exhibited by bovine type I collagen, although with smaller molecular weights observed in fish samples, which is in accordance with what is described in the literature [2].

The identification of the extracted codfish skin collagen was performed by a dot blot analysis. As shown in Figure 1B, collagen I antibody reacted with all ASColl samples, which supports the identification of codfish skin collagen as type I collagen [44]. The low intensity dots of the commercial bovine skin type I collagen sample are likely related with the degree of specificity of the antibody. According with the manufacturer, the collagen I antibody reacts with zebrafish, which explains its high reactivity with codfish samples and low reactivity with the bovine skin sample.

#### 2.1.3. Chemical Analysis

**Figure 2:**
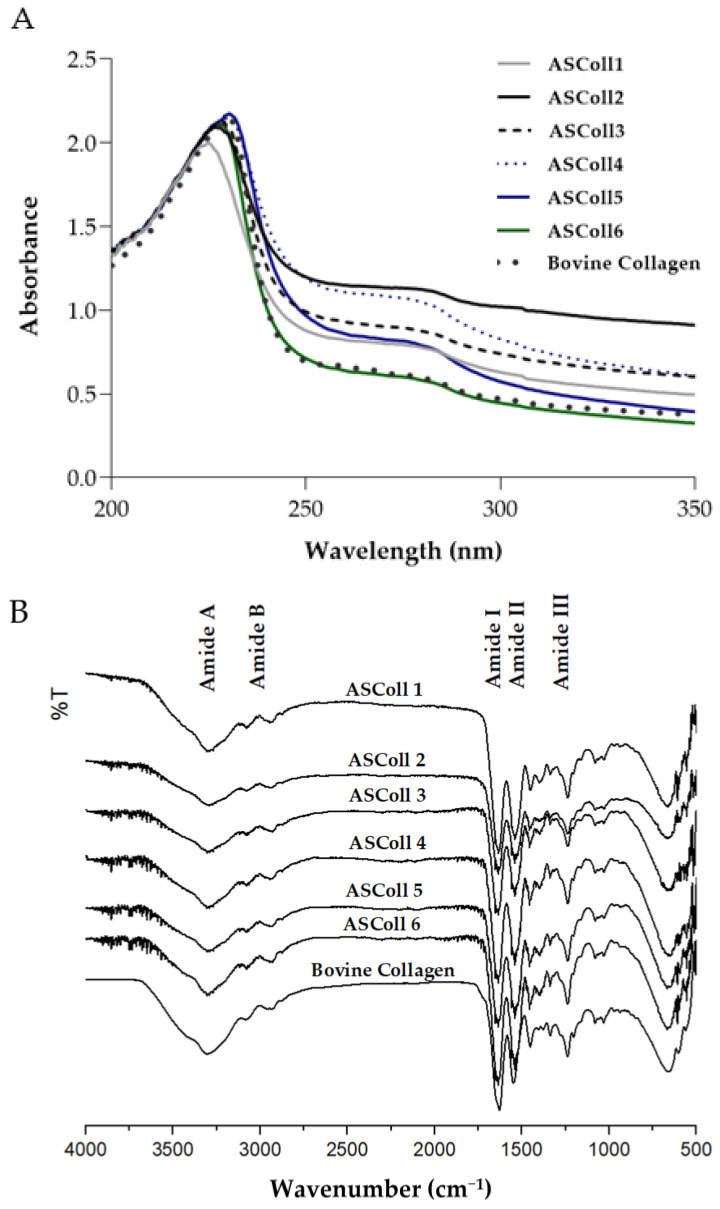
Chemical profile of ASColl. (**A**)—Absorbance spectra of the ASColl and the commercial type I collagen from bovine skin. (**B**)—Fourier transform infrared spectra of the ASColl and the commercial type I collagen from bovine skin.

To corroborate the previous analysis, a UV-Vis spectral analysis was carried out to obtain the absorbance spectra of the extracts. According to the results obtained from the extracts spectra (Figure 2A), it can be observed a maximum absorption peak between 225 nm and 230.5 nm, related with the polypeptide chains of the collagen [45]. Moreover, a broad band centered around 270 nm is detected for all extractions, due to chromophores groups of tyrosine, tryptophane and phenylalanine amino acid residues [45]. Moreover, the ASColl spectra obtained are identical to the commercial type I collagen from the bovine skin spectrum profile, which can suggest chemical similarities between samples. In this way, as stated by similar studies, the results indicate that the analyzed protein is collagen [46]. When analyzed by ATR-FTIR spectroscopy, it can be detected that the ASColl presents a chemical structure which resembles the one from bovine skin type I collagen, due to the presence of analogous chemical groups demonstrated by similar spectra (Figure 2B). For all ASColl spectra, the typical characteristic peaks for the Amide A, Amide B, Amide I, Amide II and Amide III bands linked to the collagen polypeptide chain [45,47] are present. The Amide A, that can be observed at 3392 cm^−1^, is related to the intermolecular hydrogen bonding, which presents an N-H stretching vibration [45,47]. The Amide B, which can be associated with the asymmetrical and symmetrical stretch of CH_2_ groups [45,47], can be detected at 2924 cm^−1^. Moreover, the absorption peak of the Amide I was noticed at 1635 cm^−1^, representing the stretching vibration of C=O groups of collagen [47]. Regarding the Amide II bands, which are correlated to the C-N stretching combined with N-H bending vibration [45,47], CH_2_ bending [47] and COO- symmetrical stretching [47], have occurred at 1537 cm^−1^, 1450 cm^−1^ and 1398 cm^−1^, respectively. Finally, the Amide III band, associated to N-H bending along with C-N stretching [45,47], was observed at 1336 cm^−1^, while the band correspondent to C-O stretching [45,47] was detected at 1236 cm^−1^.

#### 2.1.4. Protein Structural Analysis

**Figure 3:**
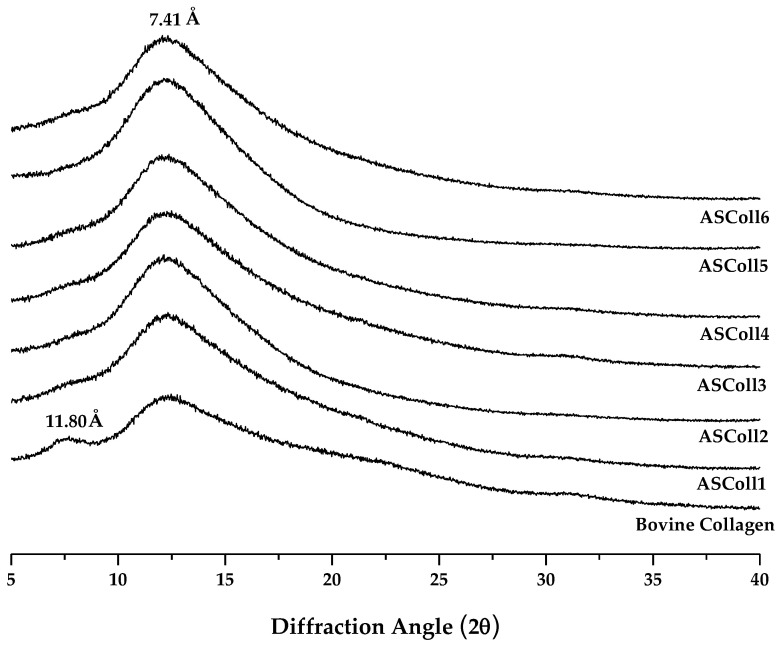
X-ray Diffraction spectra of the ASColl and the commercial type I collagen from bovine skin.

An XRD analysis was carried out to characterize the secondary structure of the ASColl [38]. As shown in Figure 3*,* the spectra for the ASColl from all six extractions, along with the spectrum from the skin type I bovine collagen, were accessed, so the differences between the organization of these collagen samples could be analyzed. Two diffraction peaks were found for the bovine collagen, namely at 7.5° and a broader one at 12°, which are characteristic of the collagen molecule [2]. For the ASColl samples obtained in all extractions, only one diffraction peak is present at 12°, coincident with the second peak from the bovine collagen, although a slight shoulder can be glimpsed at about 7.5°. The diffraction peak at 7.5° is related with the diameter of the collagen triple helix, while the diffraction peak at 12°, common to all samples, regards each α-chain [2,48,49]. Applying the Bragg law, the d values of the smaller and broader peaks were 11.80 Å and 7.41 Å, respectively. The spectrum results from the tested bovine collagen are similar to the ones obtained by Song et al. [48], which suggest that collagen was in its native conformation, while the ASColl samples were characterized by polypeptide alpha chains with a significant less amount of triple-helix.

**Figure 4:**
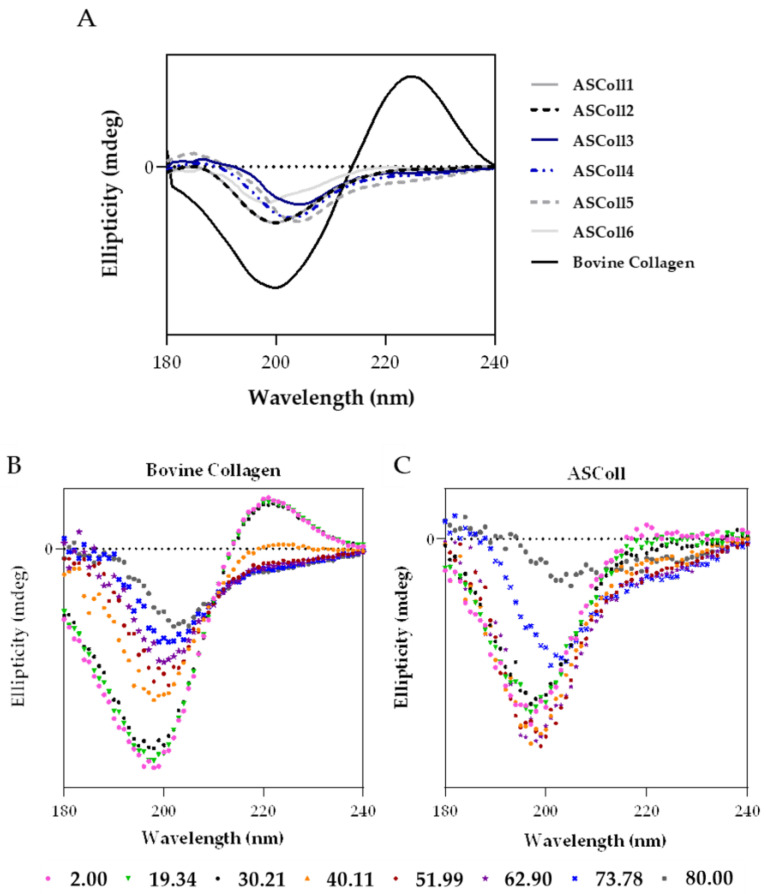
Circular dichroism (CD) analysis of ASColl. (**A**)—CD spectra of the ASColl and the commercial type I collagen from bovine skin, acquired at 25 °C. (**B**)—CD spectra of the commercial type I collagen from bovine skin, recorded as a function of temperature, between 2 °C and 80 °C. (**C**)—CD spectrum of the ASColl, recorded as a function of temperature, between 2 °C and 80 °C.

The circular dichroism spectroscopy allows the study of the proteins’ secondary structure by measuring the absorption difference between the left-handed and the right-handed circularly polarized light, defining the collagen structure [50]. This technique can also be used to determine the denaturation temperature (T_d_) of proteins, when the circular dichroism spectra is recorded as function of temperature [51]. As shown by Figure 4A, the ASColl samples presented a similar spectrum between them, being notably different from the bovine collagen spectrum. Regarding the ASColl spectra, at 25 °C, no positive peak can be observed and only a negative peak can be found, between 198 nm and 205 nm, which indicates the prevalence of random coil conformation and the absence of a triple-helix structure [51]. However, at this same temperature, the bovine skin collagen spectrum presents both a negative and positive peak, around 200 nm and 224 nm, respectively, which suggests the preservation of its native, non-denatured, structure [50,51], which corroborates the interpretations based in the XRD results.

In order to analyse the effect of temperature on the structure of collagen, and to determine its T_d_, CD spectra were acquired as function of temperature between 2 °C and 80 °C, for the commercial collagen of bovine skin (Figure 4B), used as reference, and for the ASColl (Figure 4C). The ASColl4 was selected for this analysis, since all extracts present similar spectra at 25 °C, and because the collagen from this extraction presented a lower quantity of skin pigments, being clearer and thus less prone to interferences. Regarding both bovine collagen and ASColl, at 2 °C, a positive peak can be observed at around 224 nm and 220 nm, respectively, related with the triple-helix conformation [47,52], which decreases parallelly with the increasing of temperature. In this way, the CD spectra (Figure 4B,C) shows, for both collagen samples, their loss of a triple-helix structure and overall protein organization, with the raising of temperature. To determine the T_d_ of each sample, the Boltzmann function was applied through a statistical analysis [48,53]. For the bovine collagen, the calculated temperature was 39.54 °C, while for the ASColl sample it was 22.18 °C. These results indicate a lower thermal stability of the ASColl, when compared with the analyzed commercial collagen from bovine skin, which is in agreement with other studies using other fish species [54,55]. This matter can be a barrier for the application of this molecule in a biomedical context, due to the human body’s physiological temperature [54]. Nonetheless, the T_d_ obtained for the collagen of the *Gadus morhua* skin, in this study, is higher than the collagen from other species like *Sebastes mentella* bone (17.5 °C) [56] or *P. californicus* skin (18.5 °C) [53] and similar to the T_d_ of collagen from species such as *Sticohopus vastus* integument (21.23 °C) [53] and *Brama autralis* skin (24 °C) [57]. However, it is still lower than other fish collagens, such as the one from *Cyprinus carpio* skin, scales and bones (around 28 °C) [58] and *Oreochromis niloticus* (34.5 °C) [59].

An appropriate methodology for a more in-depth analysis of the thermal behavior of collagen still needs to be implemented as the measurement is apparently highly sensitive to moisture and atmosphere.

#### 2.1.5. Collagen Cytotoxicity

**Figure 5:**
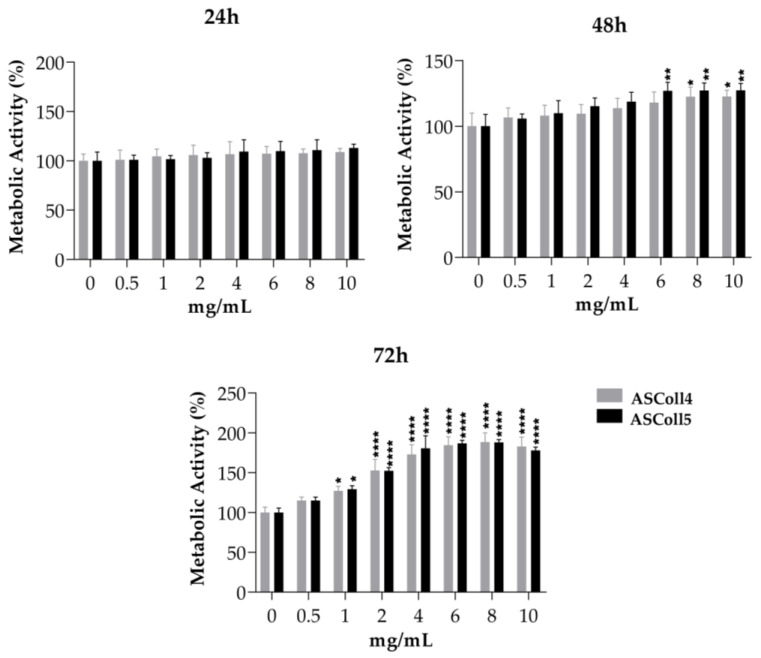
Cell viability of HaCaT keratinocytes after being cultured with different concentrations of ASColl from two different extractions (4th and 5th extraction), as measured by MTS assay. The presented values are mean ± SD of at least three independent experiments and the data was considered statistically different if *p* < 0.05, when compared with the negative control (ASColl = 0 mg/mL) by two-way ANOVA, followed by Tukey’s test. * *p* < 0.05; ** *p* < 0.01; **** *p* < 0.0001.

The cytotoxicity evaluation of the ASColl is particularly important for its biomedical and cosmeceutical application. In this way, since keratinocytes have essential functions on skin [60], the viability effect of ASColl extracts was accessed on HaCaT keratinocytes. Furthermore, to evaluate if collagen from skin batches of different years had an impact on the cellular behavior, collagen samples from the 2018 and 2019 batches were tested and compared. Since the ASColl samples presented similar characteristics on the physic-chemical characterization analysis made, the ASCol4 and ASColl5 were selected to be tested for this analysis, as representative samples from the 2019 and 2018 batches, respectively, due to being the clearest samples of each batch. As shown in Figure 5, none of the ASColl induced cytotoxicity on the tested cells, in vitro, at concentrations up to 10 mg/mL. These results are also in accordance with previous studies, which tested ASColl concentrations up to 5 mg/mL [25]. Moreover, both ASColl4 and ASColl5 significantly increased the metabolic activity of HaCaT cells, in a concentration- and time-dependent manner.

### 2.2. Membranes Developmentand and Characterization

**Figure 6:**
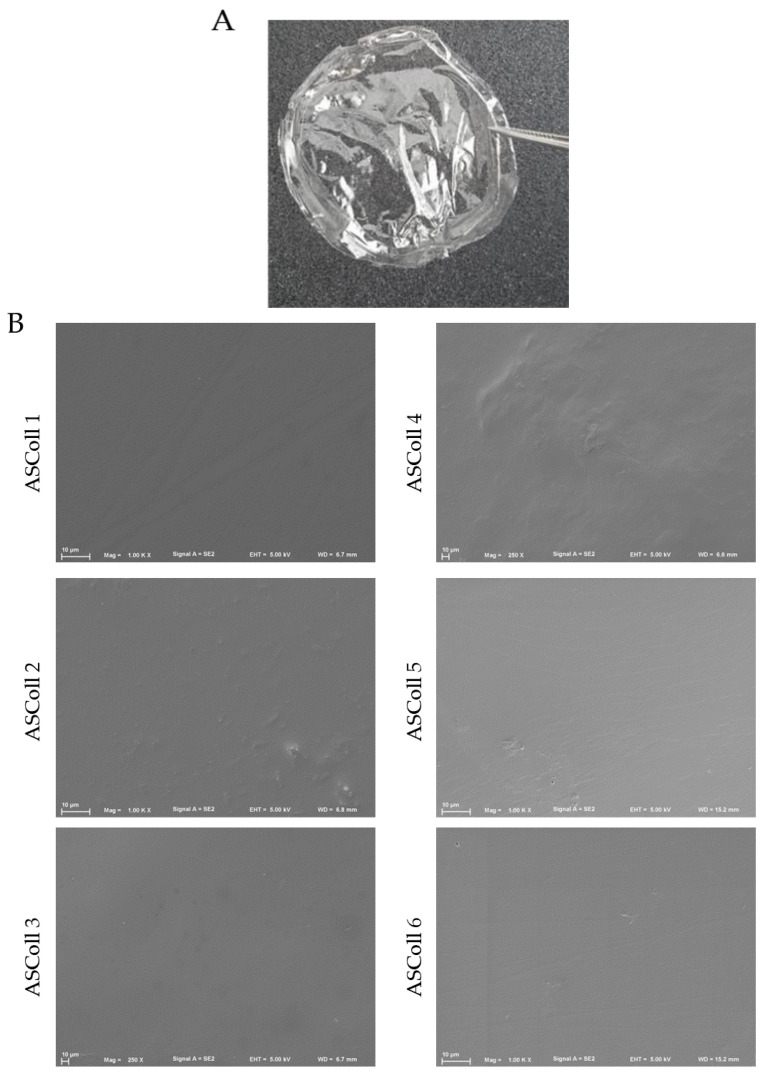
ASColl membranes morphological profile. (**A**)—Digital photo of collagen membrane developed with 1% collagen solution by applying the solvent cast method. (**B**)—Scanning electron microscopy images of the surface structure of the several ASColl membranes (1000×).

A stable and detachable collagen membrane (Figure 6A) was produced by solvent casting of ASColl solutions, as described in the Materials and Methods section. Macroscopically, the membranes were morphologically similar, transparent and colorless and, when dry, presented enough resistance to be easily handled. These characteristics are similar to other biomaterials produced by Elango and colleagues [55]. Moreover, these features can be advantageous in a biomedical context, for instance by allowing wound healing monitoring [61].

The analysis of the microscopic structure of the membranes was done to evaluate their particularities. Moreover, this was carried out to anticipate and assess their ability of cell supporting and to correlate their structure with characteristics such as fluids absorption [28,61,62,63]. For this, the microscopic surface structure of all ASColl membranes was observed through SEM (Figure 6B). By analyzing the results obtained, it can be detected that all membranes showed a compact, smooth and non-porous surface. Furthermore, no significant differences were found between membranes made with collagen from different extractions. Several non-porous collagen biomaterials, such as the Biopad [61], have been commercialized for use in biomedicine, as described in the literature. According to Tronci et al. [61], these characteristics might be helpful to oppose the excessive wound exudate absorption by the membrane, avoiding the over-drying of the wound [61,64].

#### 2.2.1. Water Contact Angle, Water Uptake and In Vitro Biodegradation

**Figure 7:**
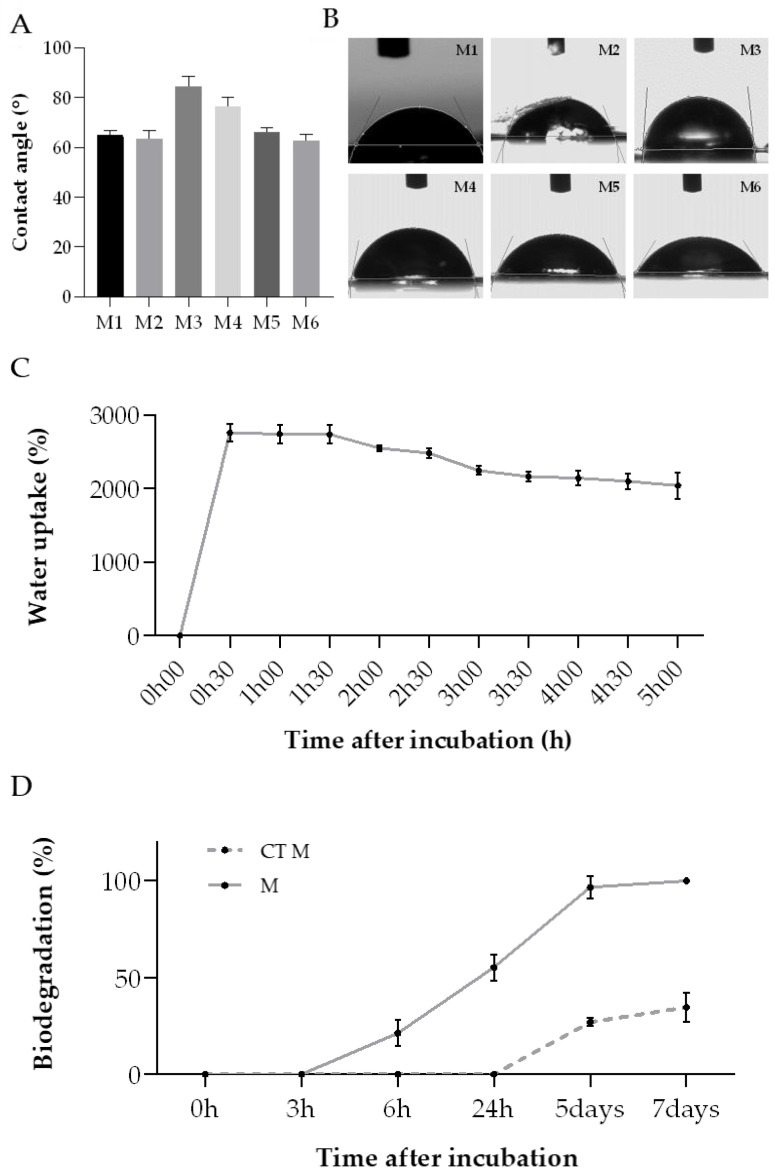
ASColl membranes physic-chemical properties analysis. (**A**)—WCA value for each membrane. (**B**)—Images of water drops on the collagen membranes. MX-membrane produced with collagen from the extraction X. (**C**)—Water uptake rate of the ASColl membranes. (**D**)—In vitro biodegradation ratio of the collagen membranes. M-membranes incubated in PBS with collagenase; CT M-membranes incubated in PBS only. The presented values are mean ± SD of three independent measurements.

As mentioned before, the degree of hydrophobicity of a biomaterial is important to determine their affinity to fluid absorption. The water contact angle (WCA) parameter can be used to assess this property, when the liquid forms an angle when in contact with the membrane, representing a boundary surface. [65]. When the water contact angle is inferior to 90°, the membrane has a hydrophilic nature; opposingly, when this parameter is above that value, the membrane is defined as hydrophobic [65,66,67]. In accordance with the obtained results (Figure 7A,B), all membranes exhibited a hydrophilic character (contact angle < 90°). This property may be helpful for wound healing applications since many commercial wound healing treatments include hydrophilic membranes, which can absorb wound exudate easily, trap moisture, and encourage cell growth [65].

The water uptake rate of all collagen membranes was measured as a function of time to further analyse the membranes’ absorption capability and corroborate the WCA results (Figure 7C). According to this, the collagen membranes demonstrated an elevated water uptake ratio, almost reaching a maximum of 2800%, which was achieved within the first 30 min of the experiment. This value decreased slightly after 1 h, ending up entering an equilibrium state upon 5 h of incubation. On the whole, these outcomes indicate that this type of behavior might be advantageous for removing the wound exudate excess, helping in the wound healing process [61].

Several enzymes, present in the human skin, participate on skin repair during the wound healing process [68]. In this way, it is needed to evaluate the performance of the developed collagen membranes when exposed to those enzymes. The in vitro biodegradation of these biomaterials is usually tested by using collagenase, which is a type of matrix–proteinase naturally present in the tissues, inclusively in skin [28,69,70]. Given that the previous analysis showed similar results for all membranes, regardless of the extraction, the membranes produced with the ASColl4 were selected for this experiment, since the collagen from this extraction presented less skin pigments and overall, less impurities, as mentioned before. Regarding the results obtained, the ASColl4 membranes that were exposed to collagenase (M) started to degrade sooner—between 3 h and 6 h after incubation—than the membranes incubated in PBS only, used as controls (CT M), which started to degrade only 24 h after incubation (Figure 7D). Moreover, 100% biodegradation was reached on the 7th day of incubation in PBS supplemented with collagenase, while in the absence of the enzyme only 34.7% of weight loss was observed at the same timepoint. This might indicate that the use of a crosslinking method is needed, in a way to improve the membranes’ enzymatic resistance, as cohesiveness and compliance with human physiologic temperature are important to assure a good performance [71,72].

#### 2.2.2. Cell Viability and Proliferation

**Figure 8:**
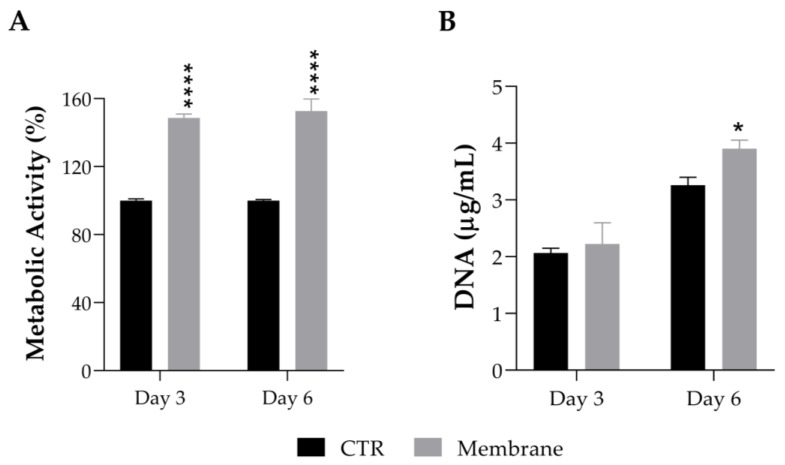
Cell viability and proliferation of HaCaT keratinocytes after being cultured with an ASColl membrane during up to 6 days. (**A**)—Metabolic activity, as measured by MTS assay. (**B**)—DNA quantification, as measured by PicoGreen assay. The presented values are mean ± SD of at least three independent experiments and the data were considered statistically different if *p* < 0.05, when compared with non-treated cells (CTR) by two-way ANOVA, followed by Tukey’s test. * *p* < 0.05; **** *p* < 0.0001.

The effect of the ASColl membranes, produced with collagen from the 4th extraction, on the cell viability of a human keratinocytes cell line (HaCaT) was assessed by MTS assay. These cells were chose to perform this evaluation because keratinocytes are the main cells that constitute the epidermis, which is the most outer layer of skin, being the primary cells to be in contact with topical formulations, such as skin patches or other biomaterials for skincare application [60]. According to the results obtained (Figure 8A), the metabolic activity of the HaCaT cells cultured with the ASColl membranes increased to 148% at day 3 after incubation, and to 152% at day 6 after incubation, when compared with the non-treated cells (CTR), with a statistically significant difference between groups for both timepoints. Considering these results, the DNA was quantified to assess the effect of the ASColl membranes on proliferation of HaCaT cells. As shown in Figure 8B, the DNA quantity increased to 2.22 µg/mL and 3.90 µg/mL at day 3 and 6 after incubation, respectively, in the cells exposed to the collagen membranes, being statistically significant in the last timepoint. According to the standard ISO 10993-5:2009(E) [73], a biomaterial is only defined as cytotoxic when cell viability is lower than 70%, when compared with the control, which does not happen with the ASColl membranes in this cell line. Indeed, the developed ASColl membranes induced an increment in both cell metabolic activity and proliferation of keratinocytes.

These results are in accordance with previous studies, using different types of collagen biomaterials [74,75,76]. As described by Zhou et al. [74] and Savencu et al. [77], the high water uptake capacity of the collagen membranes, as well as their smooth structure, is beneficial for cell growth and proliferation, which indicates that the developed ASColl membranes might be suitable for future application on skin therapeutic and care approaches.

In terms of added biological activities for biomedical and cosmeceutical application, it might be beneficial to incorporate other components along with collagen, regarding future biomaterial formulation, as is the case of some lipids also present in fish products, namely polyunsaturated fatty acids. Moreover, more tests should be done to evaluate aspects such as cell migration and the impact on wound closure, for instance.

## 3. Materials and Methods

### 3.1. Raw Materials

The skin from Atlantic codfish (*Gadus morhua*), kindly offered by Frigoríficos da Ermida Lda. (Gafanha da Nazaré, Portugal), was removed from the fish treated with salt brine. These skin samples were then brought to the laboratory and stored at −20 °C until further use. In total, six extractions were performed, using two different skin batches, one obtained in 2019 (1st, 2nd and 4th extractions) and the other obtained in 2018 (3rd, 5th and 6th extractions).

### 3.2. Collagen Extraction and Purification

The method used for the extraction and isolation of the collagen from the skin samples was adapted from Alves et al. [2] and performed at 4 °C.

The skin samples were cleaned and rinsed with distilled water to remove the salt. After removing the excess water, the samples were weighed and cut into ≈3 × 3 cm. To remove non-collagenous proteins, the samples were placed into a 0.1 M NaOH (ITW Reagents, Barcelona, Spain) solution with a 1:10 *w*/*v* ratio, under stirring for 48 h and the solution was changed three times. Then, the excess of NaOH was removed with distilled water until the pH was near 7. To extract the collagen from the samples, an acid extraction was performed. For that, the skin was grounded and placed into a 0.5 M acetic acid (Sigma-Aldrich, St. Louis, MO, USA) solution (1:10 *w*/*v*) for 72 h, under stirring. The remaining pigments were removed, and the resultant supernatant was separated from the skin by filtration using a gaze. To precipitate de ASColl, solid NaCl (ITW Reagents) was added to the solution until it reached a final concentration of 0.7 M. Next, a buffer solution of 4.6 M NaCl in 0.1 M Tris-HCl (Sigma-Aldrich) (pH 7.5), was added, obtaining a solution with a final concentration of 2.3 M NaCl in 0.05 M Tris-HCl. The collagen was left to precipitate overnight, followed by centrifugation at 20,000× *g* for 25 min, at 4 °C. The precipitate was solubilized in an equal part of 0.5 M acetic acid solution and purified by dialysis, against a 0.1 M acetic acid solution, then against a 0.02 M acetic acid solution and, finally, against distilled water until the pH was 7. All dialysis solutions were changed every day. Afterwards, it was frozen at −80 °C and later, freeze-dried and stored at room temperature (RT) until further use.

This procedure was applied for all six extractions, which were termed as follows: ASColl1—collagen from the 1st extraction; ASColl2—collagen from the 2nd extraction; ASColl3—collagen from the 3rd extraction; ASColl4—collagen from the 4th extraction; ASColl5—collagen from the 5th extraction; ASColl6—collagen from the 6th extraction.

The yield of extraction was calculated using the ratio between the weight of ASColl obtained and the wet weight of the skin used for each extraction (Equation (1)):

(1) $$\mathrm{Extraction}\;\mathrm{yield}\left(\%\right)=\frac{\mathrm{weight}\;\mathrm{of}\;\mathrm{collagen}\;(g)}{\mathrm{weight}\;\mathrm{of}\;\mathrm{wet}\;\mathrm{skin}\;(g)}\;\times \;100$$

### 3.3. Sodium Dodecyl Sulfate-Polyacrylamide Gel Electrophoresis (SDS-PAGE) Analysis

Freeze-dried ASColl from each extraction was dissolved under stirring in 0.02 M acetic acid solution until complete dissolution. Then, the samples and a commercial bovine skin type I collagen sample (Sigma-Aldrich) were mixed with Bolt LDS Sample Buffer (Life Technologies, Carlsbad, CA, EUA) and heated at 70 °C for 10 min. After sample denaturation, 40 µg of each ASColl sample and 10 µg of the commercial bovine skin type I collagen sample were loaded into separate wells of a Bolt 8% Bis-Tris Plus gel (Life Technologies). Additionally, 5 µL of protein ladder (Precision Plus Protein Dual Color Standards, Bio-Rad Laboratories, Hercules, CA, USA) was also loaded. The electrophoresis was carried out at 100 V until the dye front reached the bottom of the gel.

After electrophoresis, proteins were stained with Coomassie blue by soaking the gel in the staining solution (0.125% Coomassie Blue R 250 (Bio-Rad Laboratories), 50% methanol (Sigma-Aldrich), 10% acetic acid) for 1 h with gentle agitation, followed by the distaining solution (5% methanol, 7% acetic acid), until the protein bands are visible without background staining of the gel.

### 3.4. Dot Blot Analysis

For the dot blot analysis, 0.5, 1 and 2 µg of each sample were carefully pipetted on distinct spots of a nitrocellulose membrane (Amersham Protran 0.45 μm nitrocellulose membranes, Cytiva, Marlborough, MA, USA). For a negative control, the same amounts of bovine serum albumin (BSA) were also dotted in the membrane. After drying for 30 min at RT, the membrane was blocked for 2 h in 5% bovine serum albumin (BSA, Sigma-Aldrich) in TBS-T (20 mM Tris-HCl, 150 mM NaCl, pH 7.5 with 0.05% Tween20 (Sigma-Aldrich)). After washing in TBS-T, the membrane was incubated with the primary antibody (1:1000 in 1% BSA in TBS-T) overnight at 4 °C and then, with the secondary antibody (1:30,000 in 1% BSA in TBS-T) for 1 h at RT. The primary antibody used was the rabbit anti-collagen I antibody (1:1000, ab233639, Abcam plc, Cambridge, UK) and the secondary antibody was the goat anti-rabbit IgG, HRP-linked antibody (1:20,000, 7074, Cell Signaling Technology, Inc., Danvers, MA, USA). The blot was washed with TBS-T, incubated with the Clarity Western ECL substrate (Bio-Rad Laboratories) for 1 min and visualized using the Odyssey Fc Imaging System (LI-COR Biosciences, Lincoln, NE, USA).

### 3.5. UV-VIS Spectral Analysis

To obtain the ultraviolet absorbance spectra of the extracted collagen, along with the equivalent spectra of the commercial bovine skin type I collagen, the collagen samples were dissolved in 0.5 M acetic acid (1 mg/mL) and analyzed through UV spectroscopy. The measurements were recorded on the UV-VIS spectrophotometer (UV-1601, Shimadzu Corporation, Kyoto, Japan) from 200 nm to 350 nm, at a scanning rate of 370 nm/min. The baseline was obtained using 0.5 M acetic acid.

### 3.6. Attenuated Total Reflection-Forier Transform Infrared (ATR-FTIR) Spectroscopy

To obtain the collagen infrared spectra, the lyophilized ASColl samples and a bovine type I collagen sample, used as reference, were analyzed with the FTIR IRPrestige-21 spectrometer (Shimadzu Corporation, Japan), with an attachment of total attenuated internal reflection (ATR) in the spectral region between 500 cm^−1^ and 4000 cm^−1^. The resolution used was 2.0 cm^−1^ and each spectrum is the average of 32 scans, in transmission mode.

### 3.7. X-ray Diffraction (XRD)

The XRD measurements of the lyophilized ASColl and commercial bovine collagen samples were obtained from Bragg–Brentano diffractometer (Bruker D8 AdvanceDaVinci, Germany) at 40 kV and 40 mA, using CuKα radiation. The diffractograms were acquired with the diffraction angle (2θ) in the range of 5–35°, with a step size of 0.02°, with 1 s for each step. The average size for the crystallite was calculated using the Bragg Equation (2):

(2) $$d\;\left(Å\right)=\frac{\lambda }{2\sin\theta }$$

where d is the Interplanar Space and λ the wavelength of incident light, which in the particular case of the used radiation λ_CuKα1_ = 1.5406.

### 3.8. Circular Dichroism (CD)

The Jasco J-1500 spectropolarimeter (Jasco, Tokyo, Japan) was used to obtain the circular dichroism measurements of ASColl samples, from all extractions, as well as the measurements from the commercial bovine skin collagen. To obtain the measurements, 60 μL of each sample (1.1 mg/mL in 0.02 M acetic acid) was loaded in a 0.2 mm demountable quartz cuvette (High Precision Cell, Hellma Analytics, Müllheim, Germany) and the spectra were recorded into the spectropolarimeter (25 °C, 4 s integration, a step size of 1 nm, 3 acquisitions with a slit width of 1 nm). Additionally, to determine the denaturation temperature, the ASColl from the 4th extraction was analyzed along with the bovine collagen sample. For that, continuous ramp temperature measurements were performed by adding 60 μL of each sample, (1.1 mg/mL in 0.02 M acetic acid) in the 0.2 mm demountable quartz cuvette in a temperature-controlled module. The spectra were then recorded as a function of temperature starting at 2 °C and increasing to 80 °C (2 °C steps) and then decreasing back to 2 °C (5 °C steps) for a complete cycle [51]. The denaturation temperature was followed by the change in ellipticity at, approximately, 220 nm as a function of temperature. To determine the denaturation temperature, the Boltzmann equation was applied to the graph of ellipticity, at 220 nm, as a function of temperature.

### 3.9. Membranes Development

To develop the codfish collagen-based membranes, the extracts were dissolved in 0.5 M acetic acid solution obtaining 1% (*w*/*v*) collagen solutions. The solvent casting method was then applied, where 6 mL of the resulting solutions were casted into 5 cm diameter Petri dishes, and then left to dry, into a glass desiccator, at RT. The resulting membranes were detached from the Petri dish and stored until further use.

### 3.10. Scanning Electron Microscopy (SEM)

The characterization of the produced membranes surface morphology was carried out by scanning electron microscopy (SEM). For that, a scanning electron microscope (JSM-6010 LV, JEOL, Tokyo, Japan) was used with an acceleration voltage of 5 kV and a magnification of 1000×.

### 3.11. Water Contact Angle (WCA)

To assess the collagen membranes degree of hydrophobicity, the water contact angle on their surface was analyzed, by using a goniometer OCA 15PLUS (DataPhysics Instruments GmbH, Raiffeisenstraße, Germany). A 3 µL drop of distilled water was released on the top of the membrane and the inner angle formed between the membrane and the drop immediately after contact was measured. Three measurements in different locations were made for each membrane.

### 3.12. Water Uptake

To evaluate the water uptake parameter, aliquots of the prepared membranes were weighted and placed into individual cell strainers, immersed into 8 mL of phosphate-buffered saline (PBS) (Sigma-Aldrich) (pH 7.4) solution and incubated at 37 °C. After each timepoint, the cell strainers were dried to remove the water excess, and then weighted. The experiment was made in triplicate. The water uptake ratio was calculated applying the following Equation (3):

(3) $$\mathrm{Water}\;\mathrm{uptake}\;\left(\mathrm{wt}\;\%\right)=\frac{\mathrm{Final}\;\mathrm{membrane}\;\mathrm{weight}\;(g)\;-\;\mathrm{Initial}\;\mathrm{membrane}\;\mathrm{weight}\;(g)}{\mathrm{Initial}\;\mathrm{membrane}\;\mathrm{weight}\;(g)}\;\times \;100$$

### 3.13. In Vitro Biodegradation

In order to analyze the biomaterials in vitro biodegradation, the membranes developed with collagen from the 4th extraction were cut and distributed for tubes, obtaining 1 mg of sample per tube. Afterwards, each sample was incubated with 1 mL of PBS (pH 7.4) solution containing 12.8 ng/mL of collagenase (Sigma-Aldrich), at 37 °C. To be used as controls, this same process was applied to samples incubated only in PBS (pH 7.4) solution. After each timepoint, the PBS solution was removed, and the samples were frozen at −80 °C and then freeze-dried. The enzymatic solution was renewed every 3 days to guarantee a continuous enzymatic activity. This experiment was made in triplicate. The biodegradation ratio was determined as weight loss, applying Equation (4):

(4) $$\mathrm{Weight}\;\mathrm{loss}\;\left(\%\right)=\frac{\mathrm{Initial}\;\mathrm{membrane}\;\mathrm{weight}\;(g)\;-\;\mathrm{Final}\;\mathrm{membrane}\;\mathrm{weight}\;(g)}{\mathrm{Initial}\;\mathrm{membrane}\;\mathrm{weight}\;(g)}\;\times \;100$$

### 3.14. Biological Assessment

#### 3.14.1. Cell Culture and Experimental Conditions

HaCaT human keratinocyte (CLS Cell Lines Service GmbH, Eppelheim, Germany) cell line was cultured in Dulbecco′s Modified Eagle′s Medium (Sigma-Aldrich) supplemented with 10% of FBS (Gibco, Life Technologies) and 1% antibiotic–antimycotic solution (Gibco). The cell culture was incubated at 37 °C in a humidified incubator containing 5% CO_2_ and the medium was substituted every 2 to 3 days until an 85% confluence of cells was achieved. For experiments, HaCaT cells were seeded into 48-well plates, at a density of 15,000 cells/well, and left to adhere for 24 h.

#### 3.14.2. Cell Metabolic Activity Assay

The effect of the extracted ASColl and developed membranes on the metabolic activity of HaCaT keratinocytes was determined by the MTS assay (CellTiter 96 AQueous One Solution Cell Proliferation Assay, Promega, Madison, WI, EUA).

The ASColl from the 4th and 5th extractions was dissolved in culture medium and then added to the cells at the concentrations of 0.5, 1, 2, 4, 6, 8 and 10 mg/mL. Cells incubated with culture medium alone were used as control. After 24 h, 48 h and 72 h, cells were rinsed with PBS and incubated with a 1:5 solution of MTS in culture medium (without phenol red and FBS) for 3 h at 37 °C in a humidified 5% CO_2_ atmosphere. After that, the MTS reaction medium was transferred to a 96-well plate and the absorbance measured at 490 nm using a microplate reader (Synergy HTX Multi-Mode Reader, BioTek Instruments, Winooski, VT, USA).

The ASColl membranes, prepared with the ASColl from the 4th extraction, were cut with a surgical punch to fit the wells of 48-well plates and sterilized with UV radiation for 1 h (30 min on each side). Samples from three different membranes were placed on top of seeded HaCaT cells, with the control consisting of untreated cells. After 3 and 6 days of incubation, the membranes were removed and the procedure described for the extracted ASColl was followed.

#### 3.14.3. Cell Proliferation Assay

To evaluate the effect of the developed ASColl membranes on cell proliferation, the dsDNA of HaCaT keratinocytes was quantified as an estimative of cell proliferation by carrying out a PicoGreen assay (Quant-IT PicoGreen dsDNA Assay Kit, Molecular Probes, Eugene, OR, USA).

After 3 and 6 days of incubation, the membranes were removed, and the cells rinsed with PBS and incubated with ultrapure water for 1 h at 37 °C followed by freeze−thaw cycles to lyse the cells. Cell lysates were diluted with TE buffer and mixed with the PicoGreen reagent, according to the manufacturer’s protocol. Subsequently, fluorescence of each sample was measured at an excitation wavelength of 485/20 nm and at an emission wavelength of 528/20 nm, using a microplate reader [78].

### 3.15. Statistical Analysis

The mean and standard deviation (SD) were calculated from at least three independent experiments. The GraphPad Prism 8 (GraphPad Prism 8 for windows, GraphPad Software Inc., San Diego, CA, USA) was used to perform the statistical analysis, using the two-way ANOVA followed by the Tukey’s test, considering *p* < 0.05 significant.

## 4. Conclusions

The production of collagen—ASColl—from *Gadus morhua* skins, a by-product from the fish processing industry, using an adapted acid extraction was proven reproducible, with different extractions (assessing reproducibility of method) applied on skins obtained in different occasions (assessing batch-to-batch variability) rendering materials exhibiting equivalent physicochemical features. Moreover, the produced extracts had similarities to bovine type I collagen, widely used in life sciences research, although a slightly lower molecular weight and inferior denaturation temperature, thus resulting in partially denatured protein at room temperature.

In addition, the developed ASColl membranes were capable to uptake a remarkable amount of water and apparently enhanced the metabolic activity of keratinocytes while promoting their proliferation. The suitability of these collagen biomaterials for skincare application is thus suggested for both the biomedical and cosmeceutical fields, for instance to accelerate the wound healing process. Nevertheless, the limited structural stability in aqueous media, particularly in the presence of collagenase, might require the use of additional processing, namely using crosslinking strategies.

Overall, collagen from marine sources, in particular the collagen from the Atlantic codfish skin, has the potential to become an alternative for the commercial collagens extracted from mammalians, demonstrating important traits when incorporated in biomaterials, such as collagen membranes, for skincare application.

## Figures and Tables

**Table 1:** Collagen extraction yields.

Extraction Number	Batch (Year)	Yield (%)
1st	2019	4.30
2nd	2019	4.22
3rd	2018	3.51
4th	2019	4.80
5th	2018	4.33
6th	2018	2.87

## Data Availability

The data that support the findings of this study are included in the paper or available from the corresponding author, upon reasonable request.
